# Global, regional, and national burden of tracheal, bronchus, and lung cancer attributable to ambient particulate matter pollution among adults aged 70 and above in 1990–2021 and projected to 2044

**DOI:** 10.3389/fpubh.2025.1524534

**Published:** 2025-01-23

**Authors:** Ke-Jie He, Haitao Wang, Jianguang Xu, Guoyu Gong

**Affiliations:** ^1^Quzhou People’s Hospital, The Quzhou Affiliated Hospital of Wenzhou Medical University, Quzhou, China; ^2^The School of Clinical Medical Sciences, Southwest Medical University, Luzhou, Sichuan, China; ^3^School of Medicine, Xiamen University, Xiamen, China

**Keywords:** tracheal bronchus and lung cancer, ambient particulate matter pollution, DALYs, global burden of disease, socio-demographic index, age-period-cohort modeling

## Abstract

**Background:**

Tracheal, bronchus, and lung (TBL) cancer attributable to ambient particulate matter pollution (APMP) is a growing global health concern, particularly in individuals aged 70 and above. This study aims to evaluate past trends, identify key drivers, and project future disease burden.

**Methods:**

Data from the Global Burden of Disease Study 2021 was analyzed for TBL cancer-related disability-adjusted life years (DALYs) and mortality from 1990 to 2021, stratified by SDI regions. Statistical methods, including Joinpoint regression, age-period-cohort modeling, and decomposition analysis, were used to identify temporal trends and drivers of DALYs. Future projections were made using the Nordpred model.

**Results:**

From 1990 to 2021, global DALYs of TBL cancer due to APMP increased steadily (AAPC 0.75%). Population growth was the main driver, accounting for 79.37% of the increase, with epidemiological factors playing a varying role across regions. The highest DALY growth was observed in middle SDI regions (AAPC 2.99%), while high SDI regions saw a decline (AAPC −1.76%). Projections up to 2044 suggest a substantial increase in DALYs across all SDI regions, with the fastest growth expected among individuals aged 70–74, but DALY rates are projected to decline steadily.

**Conclusion:**

Population growth is the primary factor driving the increase in DALYs associated with TBL cancer, with significant regional disparities. Projections suggest a continued rise in disease burden, particularly in lower SDI regions, underlining the urgency for targeted public health interventions and strategies to mitigate exposure and improve healthcare outcomes for at-risk populations.

## 1 Introduction

Tracheal, bronchus, and lung (TBL) cancer remain significant contributors to the global burden of disease, leading to substantial mortality and morbidity worldwide (1, 2). Recent evidence increasingly highlights the role of environmental factors, with ambient particulate matter pollution (APMP) being one of the most pervasive and dangerous contributors to respiratory diseases, including TBL cancer (2, 3). APMP consists of a complex mixture of solid and liquid particles suspended in the air, including dust, soot, and organic chemicals, primarily generated from industrial emissions, vehicle exhaust, and other anthropogenic sources (4–6). Numerous epidemiological studies have established a strong association between long-term exposure to particulate matter and increased risks of developing disease of respiratory system (7, 8) and lung cancer (9, 10), emphasizing APMP as a critical global public health challenge.

The older adult population, particularly individuals aged 70 and above, is especially vulnerable to the detrimental effects of APMP. These demographic faces compounded risks due to their prolonged lifetime exposure to pollutants, as well as age-related declines in respiratory and immune functions (11–13). Understanding the trends and determinants of TBL cancer burden in this high-risk group is crucial, especially as urbanization and industrialization intensify, leading to heightened pollution levels in many parts of the world (14, 15). Despite these known risks, comprehensive analyses that explore the global trends of TBL cancer attributable to APMP in older adults, particularly within the context of evolving demographic and epidemiological patterns, remain limited.

This study aims to bridge the gap by utilizing data from the Global Burden of Disease (GBD) 2021 research, focusing on the analysis of disability-adjusted life years (DALYs) and mortality rates due to APMP among the TBL population from 1990 to 2021, with particular emphasis on individuals aged 70 and older. Our objective is to identify the key drivers of the observed trends, such as population aging, epidemiological transitions, and changes in pollution exposure, through advanced statistical models, including age-period-cohort analysis and decomposition techniques. Additionally, we employ the Nordpred prediction model to forecast the burden of TBL cancer up to 2044, providing valuable insights into future public health challenges.

## 2 Materials and methods

### 2.1 Overview

This study leveraged extensive data from the GBD 2021 study, curated by the Institute for Health Metrics and Evaluation (IHME). The GBD study delivers annual updates on a wide spectrum of global health metrics, including detailed information on diseases, mortality, DALYs, and associated risk factors, encompassing global, regional, and country-specific trends (16, 17). Our analysis specifically focuses on the burden of TBL cancer attributable to APMP, using data spanning from 1990 to 2021 and projecting future trends up to 2044.

### 2.2 Data sources

We extracted data on TBL cancer-related mortality and DALYs from the Global Burden of Disease Collaborative Network and GBD Study 2021 results, publicly available through the IHME’s online data visualization tool.^1^ The data we used includes detailed information on age-standardized mortality and DALYs related to TBL cancer, stratified by age group, sex, year, and SDI region. The reliability of the data is further strengthened by the GBD’s approach to standardizing disease definitions and ensuring methodological consistency across countries and regions, which allows for accurate comparisons over time.

### 2.3 Analytical methods

To investigate temporal trends, regional variations, and projections for the future burden of APMP-attributable TBL cancer, we employed the following statistical and modeling techniques:

(1) *Joinpoint regression analysis*: This approach was employed to identify significant shifts in the trends of TBL cancer-related DALYs and mortality attributable to APMP over time. By detecting points where substantial changes in the annual percent change (APC) occur, Joinpoint regression estimates the average annual percent change (AAPC) across the entire study period. We applied this method globally and across different SDI regions to pinpoint periods of accelerated or slowed growth in disease burden. This model utilizes a segmented regression approach, breaking the time period into multiple segments by identifying specific Joinpoints. Each segment is then analyzed separately, offering a more nuanced understanding of disease trends during particular intervals within the study period. Developed by the Division of Cancer Control and Population Sciences at the U.S. National Cancer Institute, the Joinpoint regression model has since become a key method for analyzing patterns in disease incidence and mortality rates, providing insights that are often missed by traditional models. The Joinpoint regression model primarily utilizes the grid search method (GSM) as its default modeling approach. In this method, the study data is divided into a grid, with each intersection representing a possible scenario for the analysis. For each scenario within specified intervals, GSM calculates performance metrics for the corresponding equations using a fixed step size to identify the optimal function. Essentially, the model employs GSM to evaluate all potential Joinpoints (points where segments of the data meet) and computes metrics such as the sum of squares errors (SSE) and mean squared errors (MSE) for each candidate. The grid point with the smallest MSE is selected as the Joinpoint. The Joinpoint regression model primarily utilizes the grid search method (GSM) for identifying statistically significant change points in the data trend. In this method, we evaluate potential change points by calculating performance metrics for regression equations. While the detailed mathematical formulation is complex, the core approach involves identifying points where the data trend demonstrates statistically significant shifts, using metrics such as the sum of squared errors (SSE) and mean squared errors (MSE).

(2) *Age-period-cohort modeling*: To analyze the separate contributions of aging, specific time periods, and birth cohorts to trends in TBL cancer incidence and mortality due to APMP, we used Age-Period-Cohort (APC) model. These models disentangle the effects of aging (the impact of increasing age on disease risk), period effects (changes in disease risk over time due to societal, environmental, and healthcare factors), and cohort effects (differences in risk among individuals born in different time periods). This method was applied globally and by SDI level to evaluate the influence of demographic shifts, healthcare improvements, and environmental changes. The age effect refers to the impact of aging on the risk of incidence or mortality from TBL cancer caused by APMP. Period effect refers to the influence of environmental, medical, and social factors on the incidence and mortality rates of TBL cancer over a specific time period. The cohort effect describes the impact of birth cohorts on the risk of TBL cancer incidence and mortality caused by APMP, representing the evolution of risk for individuals born during specific time periods. T2DM mortality estimates and population statistics from different nations and areas were used as input data for the Age-Period-Cohort (APC) model for the GBD 2021. Using this model, the overall temporal trends in mortality were calculated, which were stated as the net drift, or the annual percentage change in mortality, expressed as a percentage per year. The trend due to calendar time and the trend attributable to subsequent cohorts make up the two main components of the net drift. Additionally, the Age-Period-Cohort (APC) model offered estimates of the temporal trends in mortality within particular age groups. These estimates were expressed as the local drift, which reflected the impact of birth cohort effects, or as the annual percentage change of age-specific mortality. A change in mortality of ±1% per year is deemed significant correlating to changes of approximately ±10, ±18, and ± 26% in the mortality rate over 10, 20, and 30 years, respectively. Using a Wald chi-squared test, the significance of these yearly percentage changes was assessed. In addition, the outputs of the Age-Period-Cohort (APC) included adjusted longitudinal age-specific rates for the reference cohort to demonstrate age-related natural trends (i.e., age effects), as well as period (cohort) relative risks of mortality for each period (cohort) to indicate period (cohort) effects. We calculated relative risk by comparing age-specific rates within each cohort to those in a reference cohort, while both sets of rate ratio curves fully capture net drift. The choice of reference cohort is arbitrary and has no impact on result interpretation. To account for variability in DALY rates and model projections, we assumed that these rates followed a log-normal distribution. To estimate the uncertainty around the predicted increases in DALYs and mortality rates, we applied bootstrap resampling techniques, generating 1,000 iterations to compute 95% confidence intervals (CIs). All statistical analyses were performed using Python version 3.7.3, with statistical significance defined as *p* < 0.05. Additional details regarding these methods are provided in Supplementary material 1.

(3) *Decomposition analysis*: Decomposition analysis was carried out to determine the relative contributions of population growth, aging, and epidemiological changes (such as urbanization and shifts in lifestyle) to the rise in TBL cancer-related DALYs attributed to APMP. This analysis was performed for the global population and further broken down by SDI region, offering insight into the varying drivers of disease burden across different socio-economic environments.

(4) *Nordpred prediction model*: To forecast the future burden of TBL cancer attributable to APMP up to 2044, we used the Nordpred prediction model. This model leverages historical data to predict future trends, taking into account current and projected demographic changes, including population aging and growth, along with shifts in epidemiological risk factors. These projections provide estimates of future DALYs and DALY rates, stratified by age group, sex, and SDI level, with a particular focus on the older adult population (those aged 70 and older), where the burden is anticipated to increase the most. Regarding the quantification of uncertainties in the Nordpred model, we have employed a rigorous approach to capture the inherent variability and limitations of the data and modeling techniques. First, we have utilized the Nordpred model’s built-in functionality to calculate the 95% confidence intervals around the projected TBL cancer incidence and mortality rates. This provides a statistical measure of the uncertainty associated with the point estimates, accounting for the historical variability in the observed data. Additionally, we have conducted extensive sensitivity analyses by varying key input parameters, such as the APMP exposure levels, population projections, and cancer risk estimates. This allows us to generate a range of potential outcomes and understand the sensitivity of the results to these underlying assumptions. Furthermore, we have also incorporated uncertainty estimates from the GBD study’s estimates of APMP-attributable TBL cancer fraction, which are based on a comprehensive meta-analysis of epidemiological evidence and account for potential heterogeneity in the exposure-response relationships.

### 2.4 Socio-demographic index

The Socio-Demographic Index (SDI) is a composite indicator designed to capture the overall development level of a region or country. It is constructed using three key dimensions: income per capita, which reflects the economic prosperity of a population; educational attainment, which measures the average level of education among the population, serving as a proxy for human capital and access to knowledge; and fertility rates, which provide insight into demographic transitions and reproductive health (18, 19). Based on these components, countries and regions are categorized into five distinct SDI levels: Low, Low-middle, Middle, High-middle, and High.

## 3 Results

### 3.1 Deaths and DALYs of TBL cancer attributable to APMP in 2021

**Figure 1:**
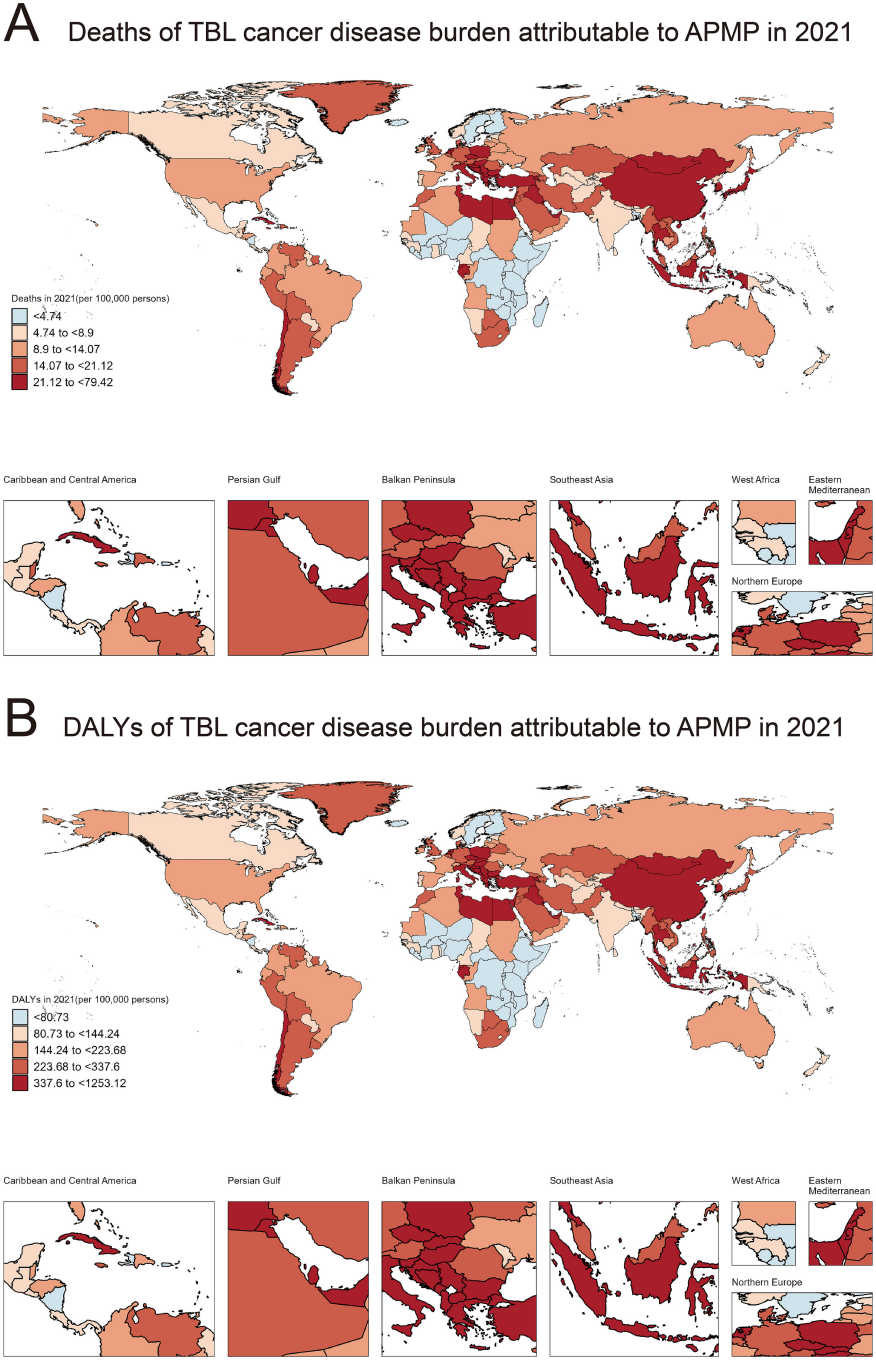
Global distribution of deaths and DALYs due to TBL cancer attributable to APMP in 2021. **(A)** Deaths of TBL cancer disease burden attributable to APMP in 2021 (per 100,000 persons). **(B)** DALYs of TBL cancer disease burden attributable to APMP in 2021 (per 100,000 persons).

In 2021, the global distribution of mortality related to TBL cancer attributable to APMP exhibited significant regional disparities (Figure 1A). Based on mortality rates, countries were classified into five categories, ranging from fewer than 4.74 deaths per 100,000 people to as many as 79.42 deaths per 100,000 people. To illustrate these disparities, we selected the top three countries in each category based on the 2021 GBD data. Countries with the lowest mortality rates (<4.74/100,000) included Kenya, Nigeria, Uganda, and Finland. Countries with slightly higher but still relatively low mortality rates (4.74/100,000–8.9/100,000) included Togo, Nepal, Tokelau, and Norway. Countries with intermediate mortality rates (8.9/100,000–14.07/100,000) included Algeria, Ireland, Brazil, and Switzerland. Higher mortality rates (14.07/100,000–21.12/100,000) were observed in countries such as Germany, the Philippines, and Pakistan, while the highest mortality rates (21.12/100,000–79.42/100,000) were found in countries such as Italy, Greece, Cuba, and China.

Similarly, the distribution of disability-adjusted life years (DALYs), reflecting both mortality and incidence rates, also demonstrated pronounced regional differences in 2021 (Figure 1B). Countries with the lowest DALYs (<80.73/100,000) included Burundi, Nicaragua, and Finland. Countries with slightly higher DALYs (80.73/100,000–144.24/100,000) included Norway, Bermuda, and Mexico. Intermediate DALYs (144.24/100,000–223.68/100,000) were reported in countries such as Brazil, Spain, and France. Countries with moderately high DALYs (223.68/100,000 to 337.6/100,000) included Jamaica, the United Kingdom, and Peru. The highest DALYs (337.6/100,000 to 1253.12/100,000) were observed in Cuba, Greece, and China. The specific information of deaths in Supplementary material 2. The specific information of DALYs in Supplementary material 3.

### 3.2 Trends in DALYs of TBL cancer attributable to APMP from 1990 to 2021 across SDI levels

**Figure 2:**
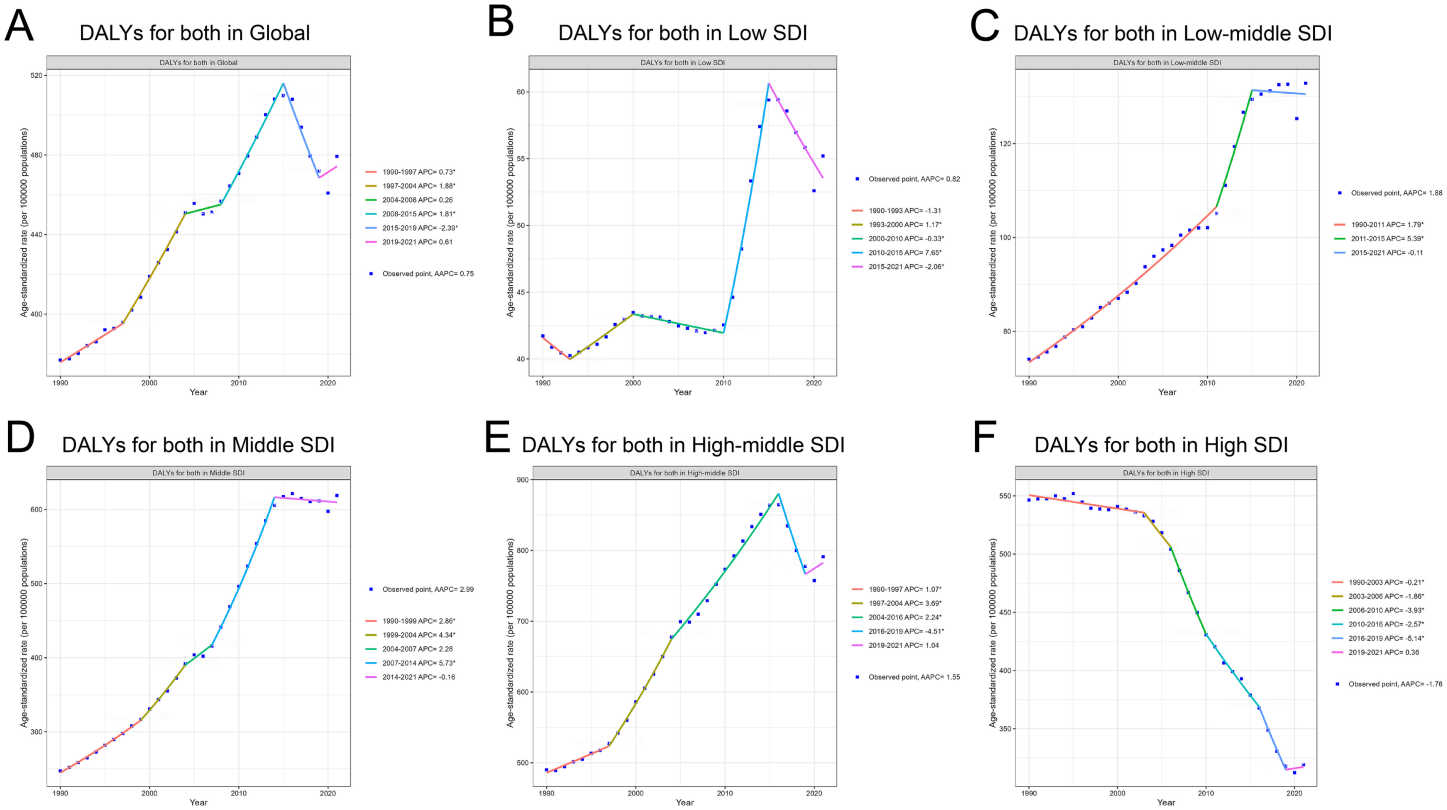
Trends in DALYs for TBL cancer attributable to APMP from 1990 to 2021 across different SDI levels. **(A)** DALYs for both in Global. **(B)** DALYs for both in Low SDI. **(C)** DALYs for both in Low-middle SDI. **(D)** DALYs for both in Middle SDI. **(E)** DALYs for both in High-middle SDI. **(F)** DALYs for both in High SDI.

From 1990 to 2021, DALYs of TBL cancer attributable to APMP steadily increased, with an AAPC of 0.75%. From 1990 to 2015, DALYs consistently rose, peaking in 2015, with the highest observed between 1997 and 2004, at 1.88%. The growth significantly slowed from 2015 to 2019 (−2.39%), but in recent years, there has been a gradual increase once again (Figure 2A).

The intensity of DALY increases varied across regions with different SDI levels. The largest growth occurred in the low-middle and middle SDI regions. In the Low-middle SDI region, DALYs continued to rise (AAPC 1.88%), with a marked acceleration between 2011 and 2015 (5.39%) (Figure 2C). The most pronounced DALY increase was seen in the Middle SDI region (AAPC: 2.99%), with the fastest growth occurring between 2007 and 2014 (Annual Percent Change 5.73%) (Figure 2D). Growth in Low and High-middle SDI regions was slower, with an AAPC of 0.82% in the Low SDI region (Figure 2B) and 1.55% in the High-middle SDI region (Figure 2E). However, it is noteworthy that the Low SDI region experienced the most rapid increase between 2010 and 2015 (Annual Percent Change 7.65%).

The High SDI region contrasted sharply with other regions, as it was the only area where DALYs showed an overall decreasing trend (AAPC −1.76%). From 1990 to 2019, DALYs consistently declined, with the sharpest decrease occurring between 2016 and 2019 (Annual Percent Change −5.14%). However, in recent years, there has been a slight upward trend, with an Annual Percent Change of 0.36% from 2019 to 2021 (Figure 2F). The specific information of AAPC in Supplementary material 4. The specific information of Annual Percent Change in Supplementary material 5.

### 3.3 Net drift in TBL cancer attributable to APMP across SDI levels

**Figure 3:**
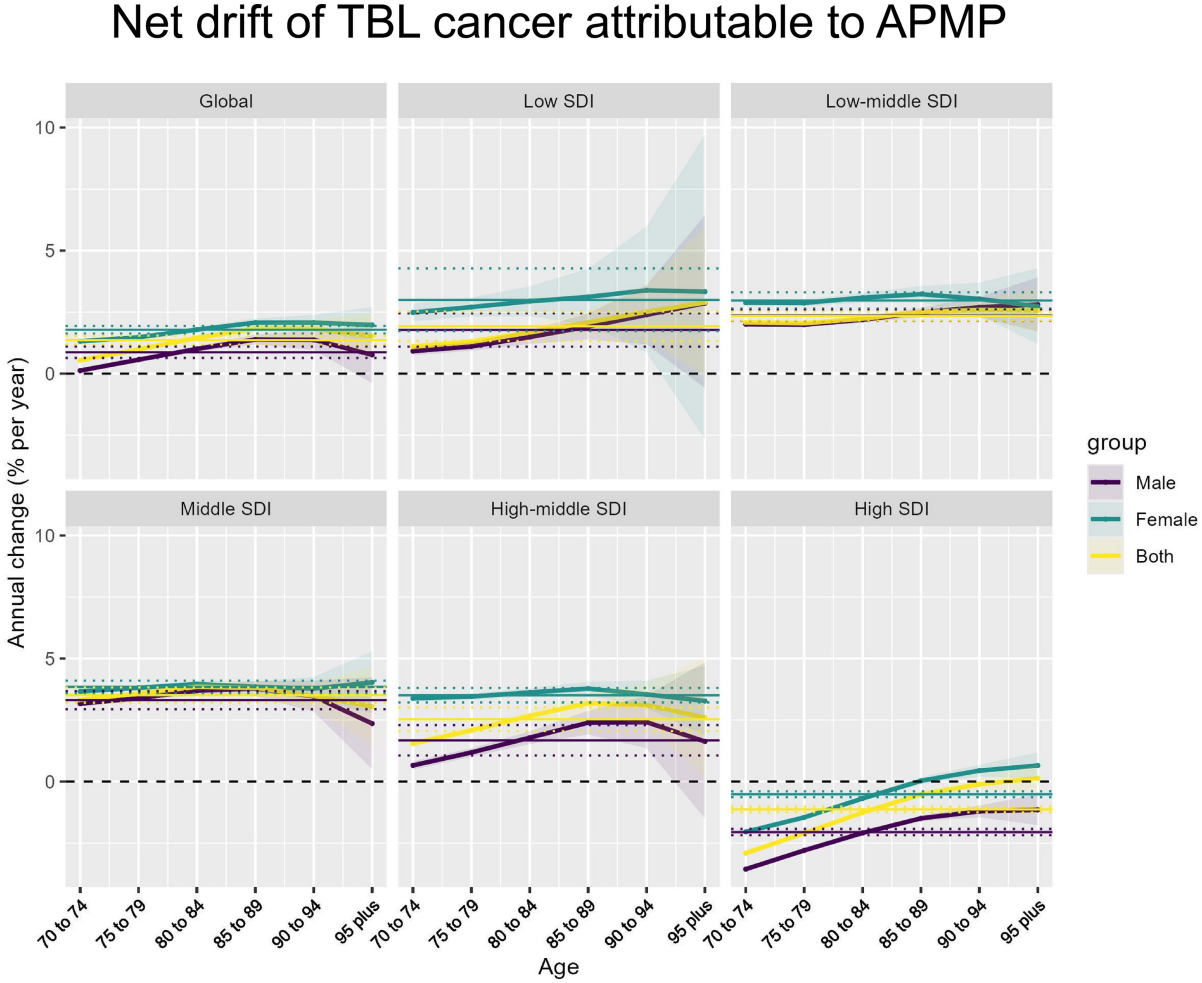
Net drift of APMP-attributable TBL cancer by SDI regions and age groups. Net annual percentage change (% per year) in APMP-attributable TBL cancer across different SDI regions and age groups, stratified by sex (male, female, and both combined).

In 2021, the global net drift in DALYs associated with TBL cancer due to APMP among individuals aged 70 and above showed significant variation across different SDI levels and between genders. Globally, the net drift for TBL cancer-related DALYs was 0.87% (95% CI, 0.64–1.10) in males and 1.78% (95% CI, 1.63–1.94) in females, indicating that the rate of increase in DALYs was higher in females compared to males. Among the various SDI levels, the Middle SDI region had the highest annual average growth rate in males, at 3.31% (95% CI, 2.94–3.68), while the High SDI region exhibited the lowest rate at −2.05% (95% CI, −2.18 to −1.92). For females, the highest growth rate was also observed in the Middle SDI region, at 3.85% (95% CI, 3.60–4.10), whereas the lowest rate was found in the High SDI region, at −0.51% (95% CI, −0.63 to −0.39). Overall, the combined annual growth rate for both males and females in the Middle SDI region was 3.51% (95% CI, 3.20–3.83), while in the High SDI region, it was −1.13% (95% CI, −1.25 to −1.02) (Figure 3). The specific information of net drift in Supplementary material 6.

### 3.4 Age, period, and cohort on TBL cancer incidence and mortality, 1990–2021

**Figure 4:**
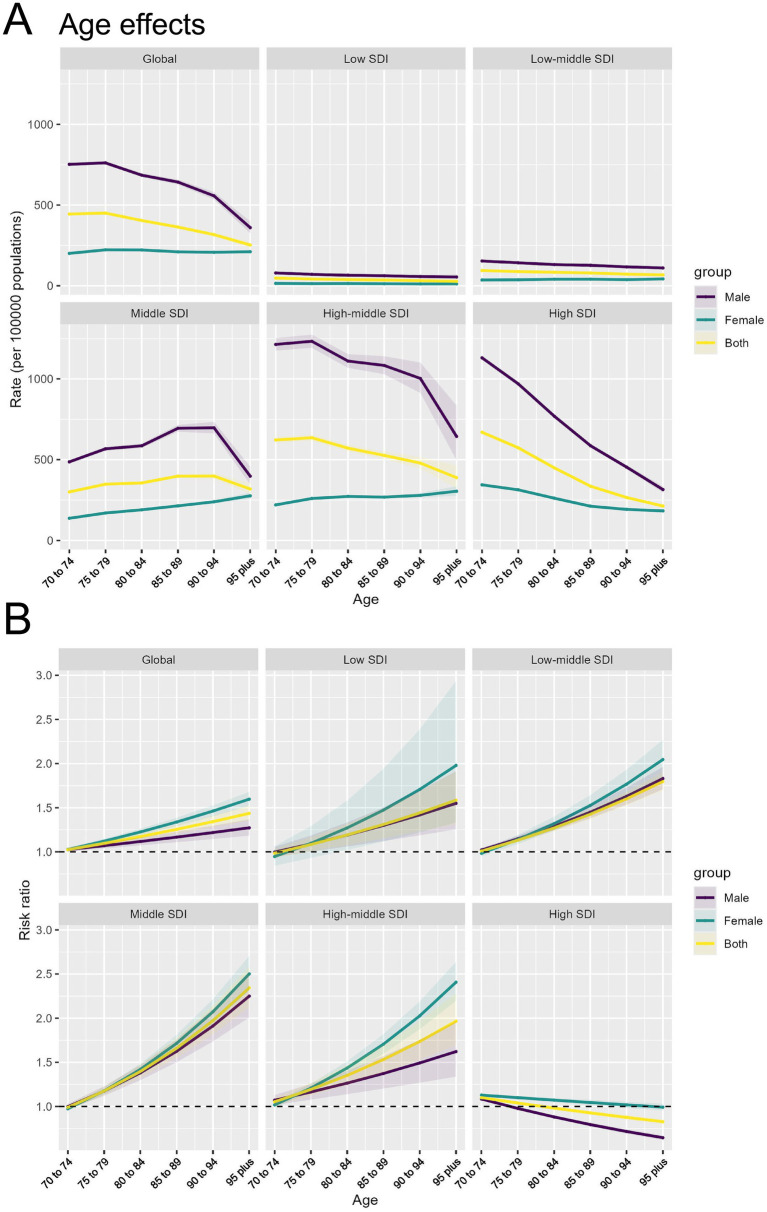
Age effects of APMP-attributable TBL cancer by SDI regions and age groups. **(A)** Age effects of APMP-attributable TBL cancer (rate per 100,000 population) across SDI regions. **(B)** Risk ratio of APMP-attributable TBL cancer across SDI regions by age groups.

**Figure 5:**
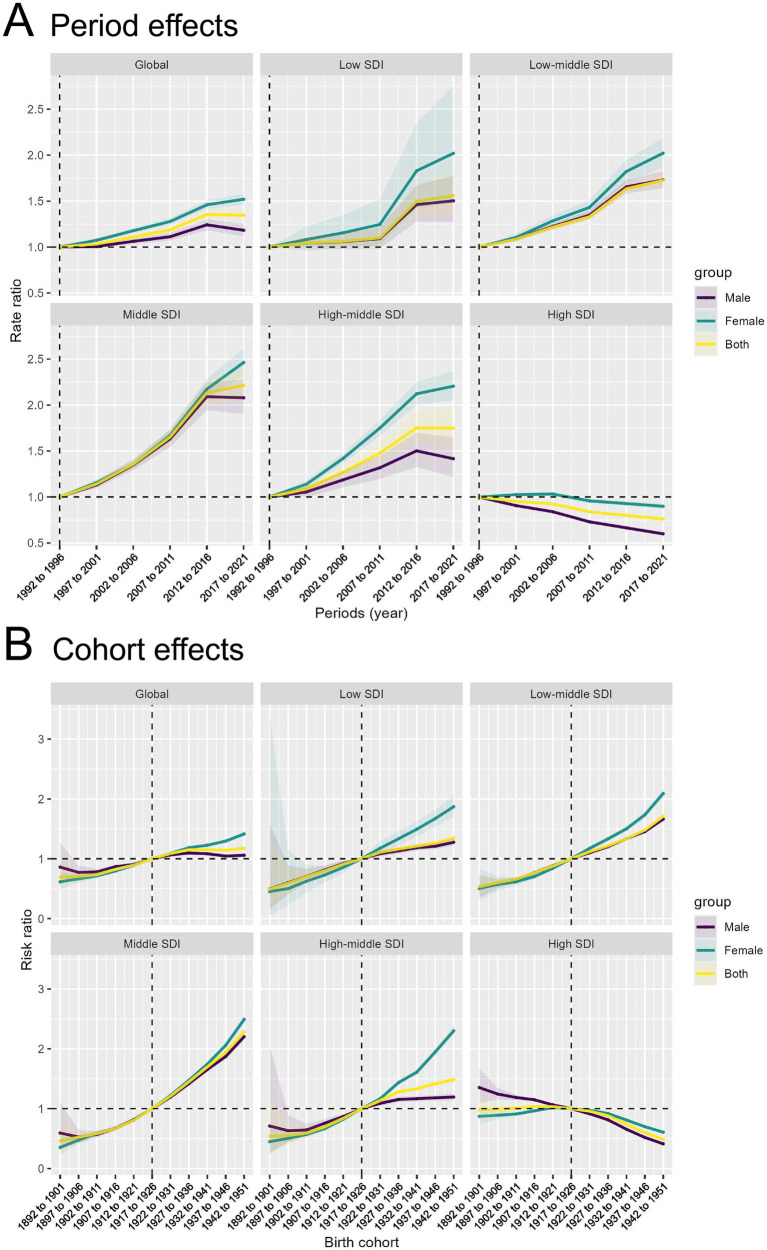
Period and cohort effects of APMP-attributable TBL cancer by SDI regions. **(A)** Period effects of APMP-attributable TBL cancer across SDI regions by periods. **(B)** Cohort effects of APMP-attributable TBL cancer across SDI regions by birth cohorts.

(1) *Age effect:* Globally and in all SDI regions except the Middle SDI region, TBL cancer incidence rates decline among individuals aged 70–95 and older, with incidence rates consistently higher in males than females across all age groups. The decline in incidence rates is slow in the Low and Low-middle SDI regions, while it is more rapid in the High-middle and High SDI regions. Notably, in the Middle SDI region, the overall incidence rate shows little change with aging, and the incidence in females increases steadily with age (Figure 4A). Globally, the risk ratio (RR) of TBL cancer attributable to APMP increases with age, with females having a higher RR than males, a trend also observed in other SDI regions. The largest increases in TBL cancer RR with age are seen in the Middle and High-middle SDI regions. However, in High SDI regions, the RR gradually decreases with age, particularly among males (Figure 4B).

(2) *Cohort effect*: Globally, the period effect indicates that from 1990 to 2021, the RR of TBL cancer attributable to APMP have consistently increased, with a similar trend observed in females. However, the RR for males have gradually declined since 2016. The most significant increase in RR is observed in the Middle SDI region, while the Low, Low-middle, and High-middle SDI regions also show a steady but slightly slower rise in RR. In contrast, the RR in High SDI regions is gradually decreasing. Across the globe and in all SDI regions, the RR for females remains higher than that for males (Figure 5A).

(3) *Period effect*: The cohort effect indicates a gradual increase in the RR of TBL cancer due to APMP, particularly in cohorts born after 1922–1931 in most SDI regions, where the RR exceeds 1. Globally and in other SDI regions, there is a general trend of increasing risk, with more recent birth cohorts (e.g., 1942–1951) showing significantly higher risk ratios than earlier cohorts (e.g., 1892–1901). However, in High SDI regions, the overall RR shows a declining trend, especially among males (Figure 5B).

### 3.5 Drivers of APMP-attributable TBL cancer, 1990–2021

**Figure 6:**
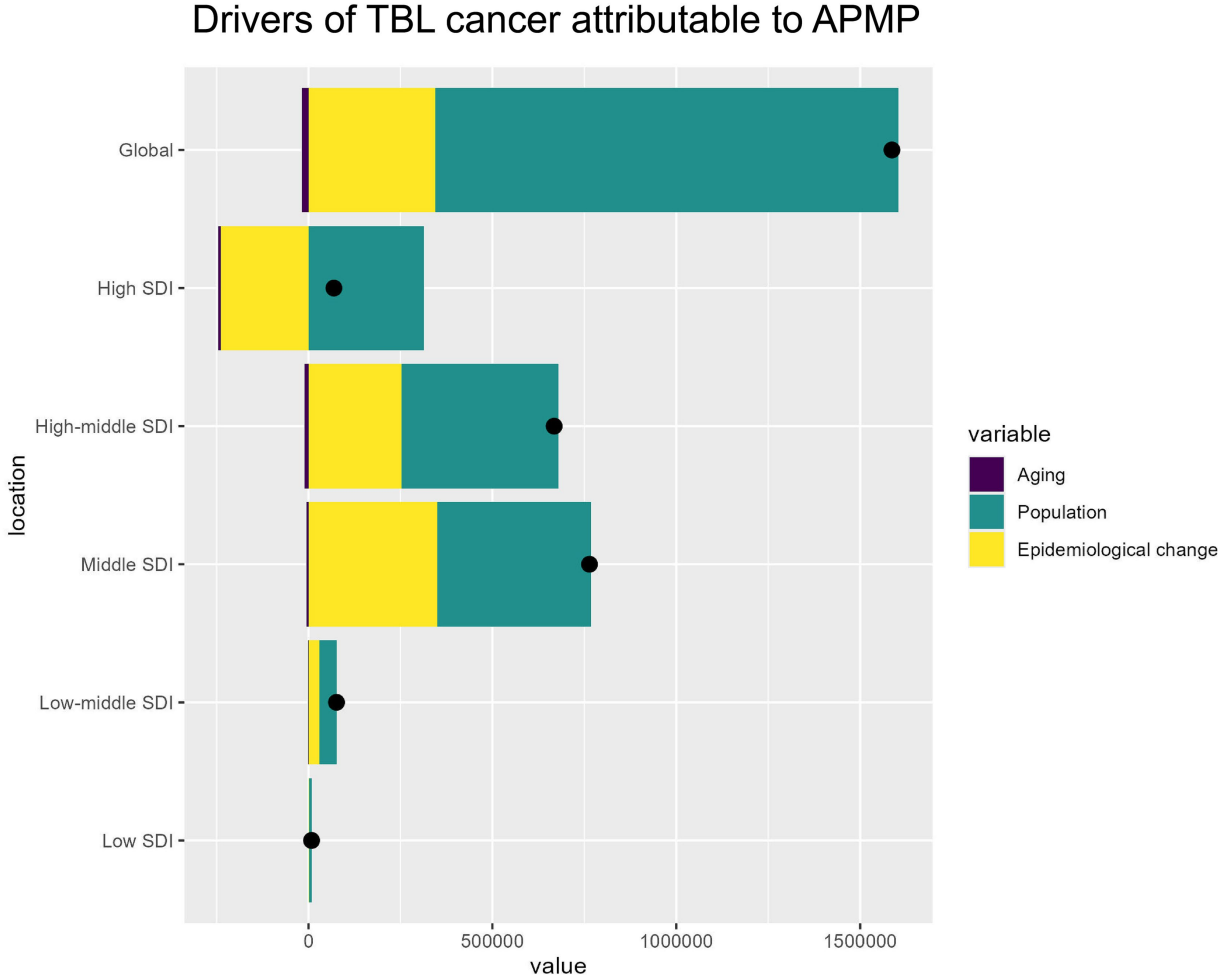
Drivers of APMP-attributable TBL cancer by SDI regions. The bar chart shows the relative contributions of aging, population growth, and epidemiological change to the overall APMP-attributable TBL cancer burden globally and across different SDI levels.

From 1990 to 2021, the increase in DALYs for TBL cancer attributable to APMP was primarily driven by population growth, accounting for 79.37% of the increase. Across different SDI regions, population growth was the dominant factor, ranging from 54.82% in the Middle SDI region to 454.47% in the High SDI region. The impact of epidemiological changes varied significantly; in most regions, particularly in the High-middle SDI (37.88%) and Middle SDI (45.8%) areas, these changes were important drivers of TBL cancer DALY increases. Conversely, in High SDI regions, epidemiological changes alleviated the burden, showing a decrease of −344.59%. The effect of aging on DALYs was minimal but consistently negative, with the greatest impact observed in High SDI regions (−9.88%). Overall, population growth emerges as the key driver of the increasing global burden of TBL cancer, while improvements in epidemiological factors have reduced the disease burden in High SDI regions (Figure 6). The specific information of driving factors in Supplementary material 7.

### 3.6 Predicted rise in APMP-attributable TBL CANCER cases and incidence rates from 1990 to 2044

**Figure 7:**
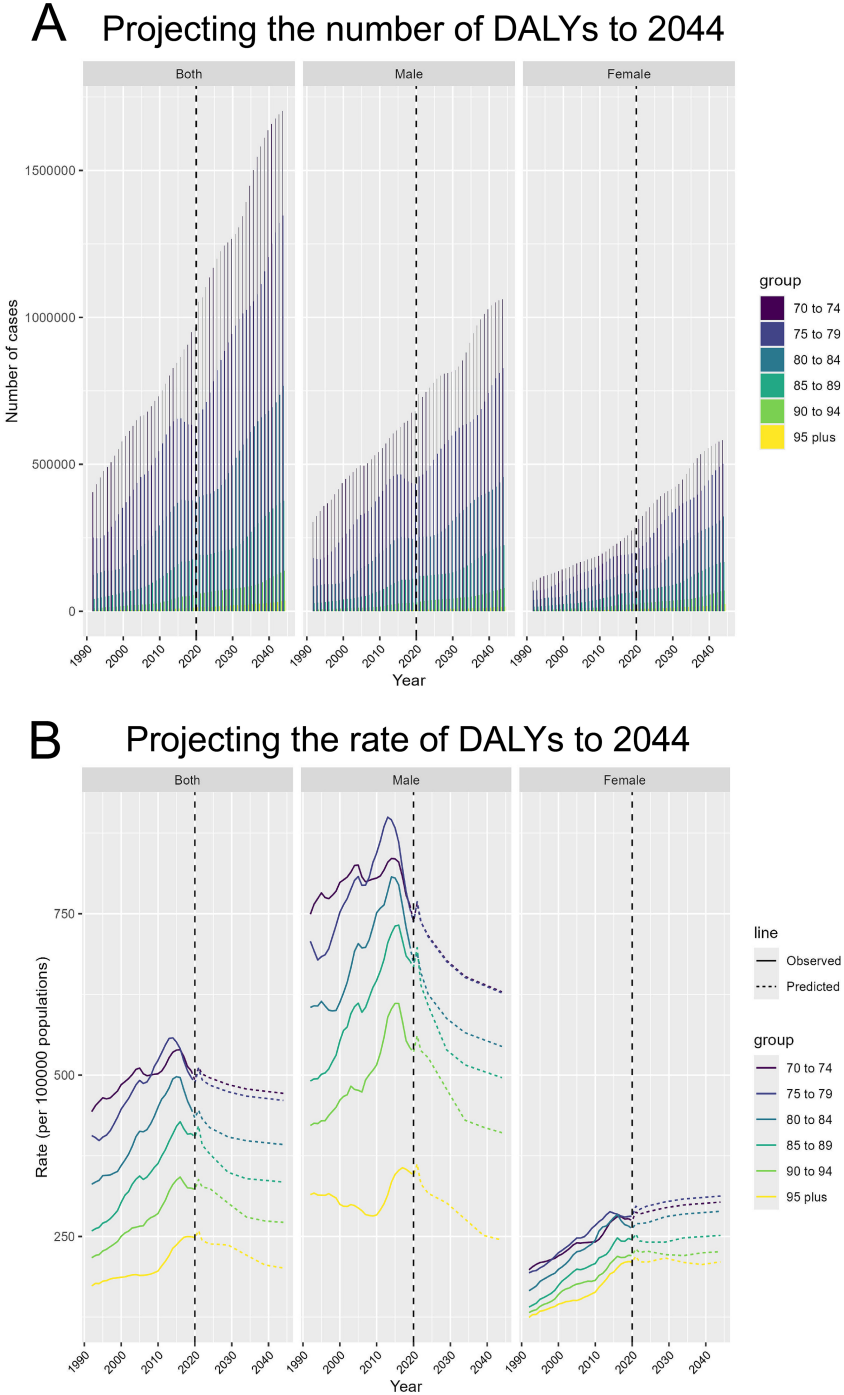
Projecting the number and rate of DALYs due to APMP-attributable TBL cancer to 2044. **(A)** Projecting the number of DALYs to 2044, by sex and age groups. **(B)** Projecting the rate of DALYs to 2044 (per 100,000 population), by sex and age groups.

Projections for DALYs attributed to TBL cancer caused by APMP from 1990 to 2044 indicate a substantial increase in DALYs for all age groups aged 70 and above. After 2020, a significant rise in DALYs is projected, with the 70–74 age group experiencing the fastest growth. Both male and female DALYs are projected to follow similar growth patterns, but the number of cases among males is anticipated to be significantly higher than that among females, particularly in younger age groups (Figure 7A). Regarding DALY rates, the projections indicate a steady decline in DALY rates across all age groups after 2020. However, for certain female age groups, such as those aged 70 to 74, the projections suggest an increase in DALY rates (Figure 7B).

## 4 Discussion

### 4.1 Global trend and regional disparities

Our analysis of the global burden of TBL cancer attributable to APMP reveals complex patterns of both decline and growth between 1990 and 2021. Globally, there was a slight decline in TBL cancer DALYs in High SDI regions (AAPC −1.76%), where improvements in healthcare infrastructure, pollution control measures, and public health awareness have likely contributed to this trend (20, 21). In contrast, Middle and Low-middle SDI regions experienced significant increases in both mortality and DALYs due to APMP, with the highest AAPC in TBL cancer burden observed in Middle SDI regions (AAPC 2.99%).

The observed regional disparities are driven by several factors. High SDI regions have benefitted from stricter environmental regulations, advancements in medical care, and public health interventions targeting both cancer prevention and air quality (22, 23). Conversely, Middle and Low SDI regions face challenges such as rapid industrialization, urbanization, and inadequate public health resources, leading to increased APMP exposure and delayed cancer diagnosis (24, 25). These disparities underscore the need for region-specific strategies in addressing TBL cancer risks, particularly in Middle and Low SDI regions, where growing urban populations are increasingly exposed to harmful pollutants.

Our analysis revealed several intriguing findings from the age-period-cohort modeling. The risk ratio (RR) of TBL cancer attributable to APMP was observed to start exceeding 1 in cohorts born after 1922–1931. This suggests that individuals in more recent birth cohorts experienced a higher risk of TBL cancer due to APMP exposure compared to earlier generations. There are a few potential explanations for this trend. First, the 20th century saw a significant rise in industrialization, urbanization, and the use of fossil fuels globally, leading to a steady accumulation of ambient particulate matter pollution. This may have disproportionately impacted the health of individuals born in more recent decades. Additionally, shifts in lifestyle and behavioral factors, such as changes in smoking prevalence, dietary patterns, and physical activity levels across birth cohorts, could have contributed to the widening disparities in TBL cancer risk, with younger generations potentially exposed to more unhealthy factors that synergize with the effects of APMP. Lastly, improvements in cancer screening and early detection methods over time may have allowed for the identification of more TBL cancer cases in more recently born cohorts, leading to a higher observed RR.

Interestingly, our analysis also revealed a gradual decline in the RR for males since 2016. This could be attributed to several factors, including the implementation of stricter pollution control measures in many regions, which may have had a more pronounced impact on reducing APMP exposure among male populations. Tobacco control programs and increasing social awareness about the harms of smoking have also contributed to declining smoking rates, particularly among men in high-income countries, which could have mitigated the synergistic effects of APMP and smoking on TBL cancer risk. Furthermore, advancements in early detection and treatment of TBL cancer may have disproportionately benefited male patients in recent years.

Our findings also highlighted significant gender-specific patterns in the TBL cancer burden. While the projected number of cases for women is significantly lower than for men, the DALY rate for women is expected to continue rising, in contrast to the declining rate for men. Several factors may contribute to these gender disparities. Women may face higher exposure to indoor air pollution due to their traditional roles in household activities, such as cooking with solid fuels, which can increase their risk of TBL cancer. Biological factors, such as hormonal differences and genetic factors (e.g., higher prevalence of EGFR mutations in women), may also influence the susceptibility of women to lung cancer development. Additionally, although smoking rates are generally lower among women, exposure to secondhand smoke can still increase their risk of TBL cancer, combined with potentially greater biological sensitivity to tobacco-related carcinogens.

### 4.2 Driving forces: population growth, aging, and epidemiological changes

The steady rise in global DALYs from TBL cancer, particularly in individuals aged 70 and older, can be largely attributed to three primary driving forces: population growth, aging, and epidemiological shifts.

Population growth emerged as the dominant factor, accounting for approximately 79.37% of the increase in DALYs from 1990 to 2021 globally. This is particularly evident in High and High-middle SDI regions, where populations have expanded rapidly in urban areas, exacerbating exposure to environmental pollutants (26, 27). Urbanization has not only increased the concentration of particulate matter but has also led to higher risks of developing chronic diseases, including TBL cancers, due to the co-occurrence of unhealthy lifestyles such as smoking, poor diets, and reduced access to healthcare (28).

Aging plays a more nuanced but significant role in shaping the disease burden. As the global population ages, the number of individuals at higher risk for TBL cancer naturally increases, particularly in the older adult population where physiological vulnerabilities to both carcinogens and APMP are heightened (29, 30). However, while aging contributes to a growing number of TBL cancer cases, its impact is less pronounced compared to population growth.

Epidemiological changes, including shifts in lifestyle, improvements in healthcare, and variations in pollution exposure, vary widely across regions. For instance, High SDI regions have seen reductions in smoking rates, better healthcare access, and cleaner air due to effective policies, contributing to the declining TBL cancer burden (31, 32). Conversely, Middle SDI regions, where economic growth has often come at the expense of environmental health, continue to see rising TBL cancer rates due to increased APMP exposure and delayed access to cancer treatment (33).

### 4.3 Future projections

The projections up to 2044 indicate a continued global increase in the burden of TBL cancer cases attributable to APMP, particularly among the 70–74 age group, although the global DALY rate is decreasing. Notably, these trends exhibit significant gender differences, with the predicted number of cases for women being significantly lower than for men. However, in contrast to the declining DALY rate for men, the DALY rate for women is expected to rise, a disparity that warrants deeper consideration. Firstly, women’s societal role in household cooking increases their exposure to solid fuels (such as wood and coal) and cooking fumes (34). Secondly, while the smoking rate among women is lower, secondhand smoke is a potential risk factor for lung cancer, and women may be more sensitive to specific carcinogens in tobacco (35). Additionally, the prevalence of EGFR mutations, which are highly associated with lung cancer, is higher in women (36). Lastly, higher estrogen levels may also make women more susceptible to lung cancer. Due to changes in social environments and dietary patterns, more women are experiencing earlier menarche and later menopause, resulting in relatively higher estrogen levels, which could also contribute to the continued rise in the DALY rate among women (37).

Accurately predicting the future trajectory of APMP levels and associated health impacts is inherently challenging, as it requires making assumptions about the pace and scale of technological and policy changes, as well as their real-world effectiveness in reducing pollution exposure. Incorporating these factors into our current modeling approach would involve a high degree of uncertainty, as the adoption and impact of clean energy solutions and air quality regulations can be influenced by a range of socioeconomic, political, and logistical factors. Furthermore, the complex interactions between APMP exposure, other risk factors (e.g., smoking, occupational exposures), and cancer development make it difficult to reliably quantify the precise reductions in TBL cancer burden that may result from future air pollution mitigation efforts. The epidemiological evidence linking APMP to cancer outcomes is still evolving, and there may be heterogeneity in the exposure-response relationships that could affect the projected impacts of clean air interventions. Given these limitations, we have chosen to discuss the potential for clean air interventions to mitigate future TBL cancer risk in a more conceptual manner, rather than attempting to incorporate specific scenarios or quantify their effects. This approach allows us to acknowledge the importance of these emerging factors, while maintaining the transparency and robustness of the core projections presented in the manuscript.

### 4.4 Limitations and future directions

#### 4.4.1 Socioeconomic diversity and limitations of the SDI grouping

While the SDI is a useful tool for categorizing countries based on developmental metrics, it has limitations when examining health outcomes across diverse socioeconomic contexts. The SDI categorizes countries into broad categories, but there can be significant differences between countries within the same SDI region, and even substantial disparities within a single country. For instance, large heterogeneous nations such as China, India, and the United States exhibit marked differences in healthcare accessibility, public health infrastructure, and economic conditions across different regions (38, 39). All these factors influence APMP exposure and TBL cancer outcomes. Future studies should incorporate more specific socio-economic indicators, such as income inequality, healthcare access, and urbanization rates, to better capture the heterogeneity within SDI categories. Additionally, local environmental policies and their enforcement should be examined as potential modifiers of TBL cancer risks attributable to APMP, particularly in Middle and Low SDI regions.

#### 4.4.2 Uncertainty of future projections and the role of technological advancements

Although the Nordpred model provides robust projections of future TBL cancer burden, inherent uncertainties exist, particularly regarding future demographic and epidemiological changes. Technological advancements, such as improvements in pollution control technologies (40), medical treatments, and early cancer detection methods (41), may significantly alter future trends in ways that are difficult to predict today.

Moreover, emerging innovations in clean energy, such as electric vehicles and renewable energy sources, have the potential to dramatically reduce APMP levels, particularly in urban environments (42). If widely adopted, these technologies could lead to significant declines in TBL cancer cases attributable to APMP, even in regions currently experiencing rapid industrialization and urbanization.

Lastly, advances in healthcare, particularly in cancer diagnosis and treatment, could mitigate the future disease burden. Precision medicine, immunotherapies, and improvements in public health surveillance could enable earlier detection and more effective management of TBL cancers, particularly in older adult populations who are most vulnerable to the disease (43). However, the equitable distribution of these advancements remains a critical challenge, particularly in Middle SDI region where healthcare disparities are pronounced.

## 5 Conclusion

In conclusion, the global burden of TBL cancer attributable to APMP is projected to continue rising, particularly in Middle and Low-middle SDI regions, driven by population growth, aging, and ongoing environmental challenges. While High SDI regions have seen improvements in managing this burden, significant regional disparities persist, underscoring the urgent need for global and region-specific interventions. Policymakers must prioritize strategies that reduce pollution exposure, improve healthcare access for older adults, and leverage technological advancements to mitigate future disease risks. Reducing the burden of TBL cancers will require concerted efforts across sectors, including environmental health, healthcare, and technological innovation, to protect the most vulnerable populations in an increasingly polluted world.

## Data Availability

The original contributions presented in the study are included in the article/Supplementary material, further inquiries can be directed to the corresponding author.

## Footnotes

^1^ https://vizhub.healthdata.org/gbd-results/
